# Supplementary figures and images for: KDM2A Deficiency in the Liver Promotes Abnormal Liver Function and Potential Liver Damage
Source: Biomolecules. 2023 Sep 27;13(10):1457. doi: 10.3390/biom13101457 (PMC10604476; doi:10.3390/biom13101457)

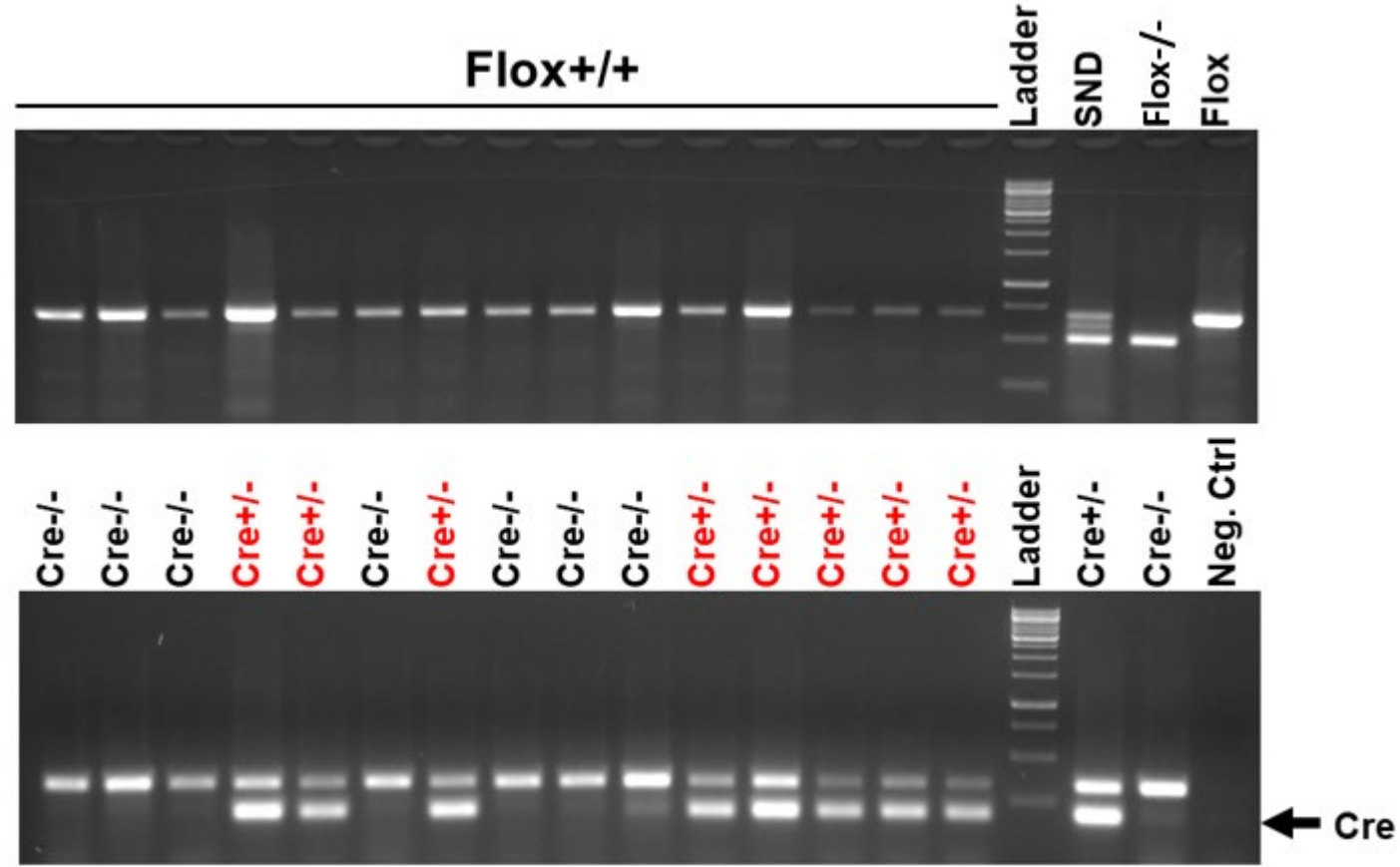

Figure S1

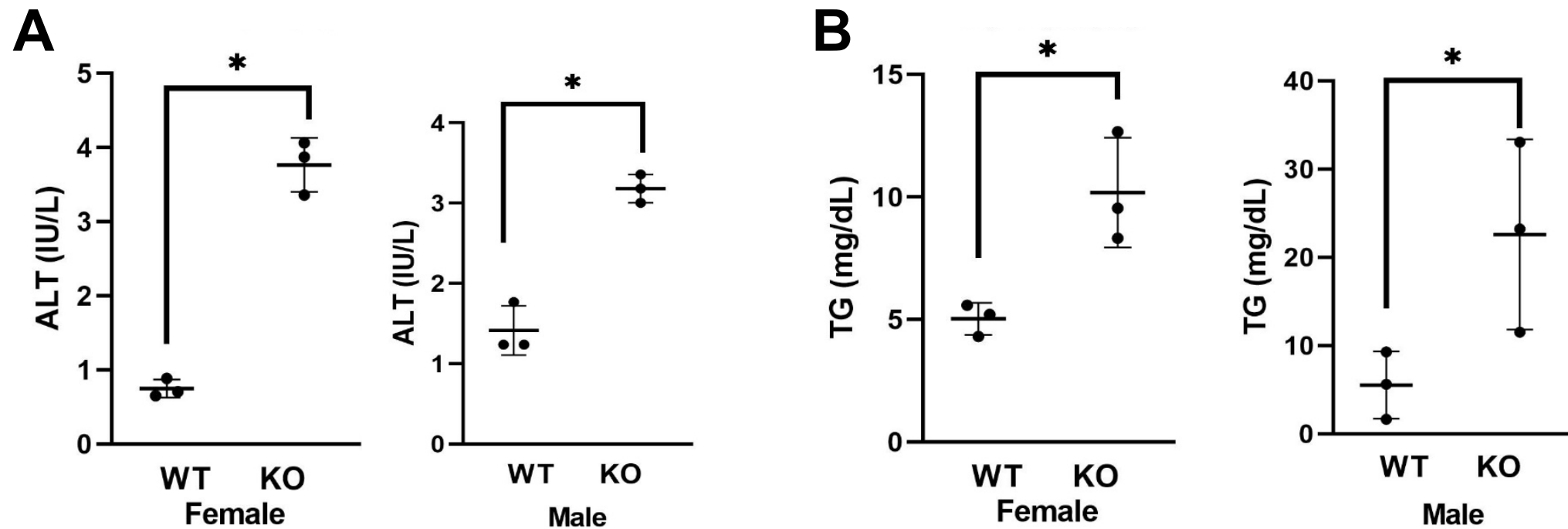

**Figure S2**

Supplement: Supplementary file 1 [file biomolecules-13-01457-s001.zip › biomolecules-2603628-supplementary.pdf]
